# Period prevalence of SARS-CoV-2 infections and willingness to vaccinate in Swiss elite athletes

**DOI:** 10.1136/bmjsem-2022-001330

**Published:** 2022-06-29

**Authors:** Michael Johannes Schmid, Merlin Örencik, Boris Gojanovic, Jürg Schmid, Achim Conzelmann

**Affiliations:** 1 Institute of Sport Science, University of Bern, Bern, Switzerland; 2 La Tour Sport Medicine, Hôpital de La Tour, Meyrin, Geneva, Switzerland; 3 Interdisciplinary Center for Adolescent Sports Medicine, Woman-Mother-Child Department (DFME), Lausanne University and CHUV Hospital, Lausanne, Switzerland

**Keywords:** Vaccination, COVID-19, Elite performance, Infection

## Abstract

**Objectives:**

(1) To assess the period prevalence of SARS-CoV-2 infections and willingness to vaccinate in Swiss elite athletes and (2) to evaluate whether sociodemographic and sport-related characteristics were associated with infection of SARS-CoV-2 in athletes.

**Methods:**

A total of 1037 elite athletes (M_age_=27.09) were surveyed in this cross-sectional study. They were asked whether they had been infected with SARS-CoV-2 and whether they would like to be vaccinated. Characteristics of a possible COVID-19 illness were also recorded.

**Results:**

During the first year of the pandemic, 14.6% of all Swiss elite athletes were found to be infected with SARS-CoV-2, and 5.4% suspected that they had been infected. Male athletes, young athletes and team sports athletes had an increased likelihood of being infected with SARS-CoV-2. There was considerable heterogeneity in the duration and severity of a COVID-19 illness in athletes. Overall, 68% of respondents indicated a willingness to be vaccinated if they were offered an opportunity to do so.

**Conclusion:**

In the first year of the pandemic, Swiss elite athletes were tested more often positive for SARS-CoV-2 than the general Swiss population. Because COVID-19 illness can impair health for a relatively long time, sports federations are advised to motivate athletes to be vaccinated.

**What is already known**

- One year after the outbreak of the COVID-19 pandemic, the period prevalence in Switzerland was estimated at 8–25%.
- The severity of the initial SARS-CoV-2 infection is most often mild in athletes.

**What are the new findings**

- During the first year of the pandemic, 14.6% of all elite athletes in Switzerland were found to be infected with SARS-CoV-2, and 5.4% suspected that they had also been infected.
- There is considerable heterogeneity in the duration and severity of COVID-19 illness in athletes.

## Introduction

Since the beginning of the COVID-19 pandemic, over 540 million SARS-CoV-2 cases were confirmed.^1^ The first COVID-19 case in Switzerland was reported by the Federal Office of Public Health on 25 February 2020.^2^ On 16 March 2020, the Federal Council of Switzerland declared public health emergency.^3^ One year after the outbreak of the COVID-19 pandemic, there were between 6 and 7 laboratory-confirmed cases (PCR tests) per 100 inhabitants.^4^ In the same period, it was estimated by means of seroprevalence studies that 8%–25% of the Swiss population have been infected with the virus.^5^

While the severity of the initial infection is most often mild and without complications in athletes, the main concerns about returning to sports and performing are linked to two conditions: pericardial and myocardial inflammation and persistence of performance-limiting symptoms.^6^ In the UK, 14% of infected athletes reported symptoms for >28 days, and 21% had a training/competition time loss of >28 days.^7^

Because of the potentially serious consequences of COVID-19, the prevalence of the disease among athletes is of major importance. Furthermore, it raises the question whether all athletes are equally affected. Thus, the aim of the current study was twofold: (a) to assess the period prevalence of SARS-CoV-2 infections and willingness for vaccination in Swiss elite athletes and (b) to investigate potential associations of sociodemographic and sport-related characteristics with infection of SARS-CoV-2.

## Methodology

### Participants

All Swiss elite athletes who were members of the national team in their sport were invited for participation (N=2838, M_age_=27.53, SD=8.55, 38.4% female, 61.6% male). Data of 1037 athletes (36.5%), who fully completed the questionnaire, were included in the data analysis M_age_=27.09, SD=8.86, 42.7% women, 57.3% men. They trained on average 14.41 hours per week (SD=7.38) and had 13.78 competition days (SD=19.69) in 2020. To examine possible selection biases, participating individuals (n=1037) were compared with those who did not (n=1801) on demographic and sports-related characteristics. The logistic regression model containing five independent variables was significant, χ^2^=94.868, *N*=2838, df=5, *p*≤0.001, R^2^ (Nagelkerke)=0.045. Specifically, there was a slight over-representation of female athletes, athletes from winter sports, athletes from individual sports, young athletes and successful athletes. This could be because these athletes benefit more from our collaboration institutions (Swiss Sports Aid Foundation, Swiss Olympic Association) and, therefore, felt particularly motivated to complete the questionnaire. Analyses of the technical data of the survey revealed that no respondent chose to discontinue the survey in the immediate vicinity of the items relating to COVID-19.

### Measures

The questionnaire (in German and French) explored the current life situation (ie, sport training volume, education, vocation), including health. Particularly, we asked the participants whether they had been infected with SARS-CoV-2 (*yes, with positive test result; yes, probably, but without a positive test result; no*). Subsequently, the infected athletes rated the severity of COVID-19 symptoms and the influence on their athletic performance. Moreover, infected individuals were asked whether they had recovered and how many days COVID-19 had affected their athletic performance. The correlation between the three indicators ranged from 0.40≤*r*_s_≤0.54. Finally, the willingness to be vaccinated against COVID-19 on vaccination availability was surveyed.

### Procedures

The athletes were surveyed with an online questionnaire 1 year after the outbreak of the COVID-19 pandemic in Switzerland (March 2021).

### Data analysis

We used frequency tables to analyse period prevalence of a SARS-CoV-2 infection and vaccination willingness. Results for severity of illness, limitation and duration of illness are presented as mean and SD. Furthermore, we examined whether gender, age, type of sport (individual vs team, summer vs winter) and training volume are associated with an increased risk of a SARS-CoV-2 infection. We calculated period prevalence as the number of infected participants divided by the total number of participants and performed logistic regressions to estimate ORs (and 95% CIs) for the respective associations between gender, age, type of sport and training volume with the dependent variable (SARS-CoV-2 infection). A 5% significance level was chosen for statistical testing. All analyses were conducted using IBM SPSS Statistics (V.28).^8^

## Results

There was an observed period prevalence of 14.6% across the entire sample (see table 1). However, the total period prevalence is likely to be higher, as 5.4% suspected that they also had the disease but had not been tested at the time.

**Table 1:** Period prevalence of SARS-CoV-2 infections in Swiss elite athletes by gender

SARS-CoV-2 infection status	Female athletes		Male athletes		Overall	
	n	%	n	%	n	%
Infected athletes	48	10.8	103	17.3	151	14.6
Probably infected athletes	22	5.0	34	5.7	56	5.4
Non-infected athletes	373	84.2	457	76.9	830	80.0
Overall	443	100.0	594	100.0	1037	100.0

Infected athletes were tested positive on SARS-CoV-2; probably infected athletes assumed to have been infected but were not tested.

**Figure 1:**
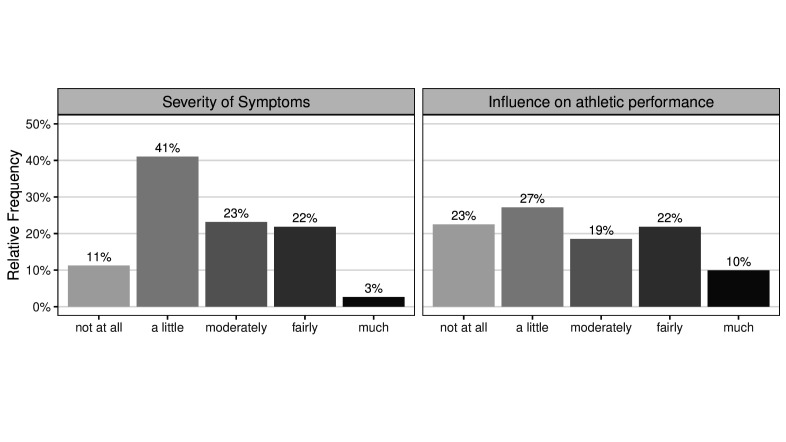
Severity of symptoms and influence on athletic performance of a SARS-CoV-2 infection among Swiss elite athletes (n=151).

The majority of athletes (75.6%) who were diagnosed with COVID-19 (n=151) had mild to moderate symptoms, and COVID-19 had mild to moderate adverse effects on athletic performance in 68.2% of the athletes (see table 2 and figure 1). Based on the median, the illness affected the athletic performance for 10 days on average. However, there is notable heterogeneity regarding severity and influence on performance. For example, one person stated that he or she had already been experiencing negative effects on athletic performance for 360 days. Overall, nine athletes (6.0% of all infected athletes) indicated that they were struggling with reduced performance for 12 or more weeks after onset of the illness.

**Table 2:** Severity of symptoms, influence on athletic performance and duration of negative effects of a SARS-CoV-2 infection among Swiss elite athletes (n=151)

Consequences of SARS-CoV-2 infection	Female athletes						Male athletes						Overall					
	M	SD	Mdn	IQR	Min.	*Max.*	M	SD	Mdn	IQR	Min.	Max.	M	SD	Mdn	IQR	Min.	Max.
Severity of symptoms	1.65	1.16	2	2	0	4	1.63	0.97	1	1	0	4	1.64	1.03	1	1	0	4
Influence on athletic performance	1.75	1.26	1	2	0	4	1.67	1.33	2	3	0	4	1.70	1.31	2	2	0	4
Fully recovered from COVID-19																		
Duration of negative effects	9.75	9.46	9	12	0	35	13.04	14.07	10	18	0	70	12.03	12.88	10	15	0	70
Not yet recovered from COVID-19*																		
Duration of negative effects (ongoing)	35.25	49.01	12	58	4	160	74.82	81.35	50	106	1	360	60.85	73.35	27	80	1	360

Scale severity of symptoms: 0=*not at all*, 1=*a little*, 2=*moderately*, 3=*fairly*, 4=*much*; scale influence on performance: 0=*not at all*, 1=*a little*, 2=*moderately*, 3=*fairly*, 4=*much*; duration of negative effects: numerical value.

A total of 29 individuals (19.2%) of all infected persons (n=151) stated that they had not yet regained their normal athletic performance. Therefore, we asked how long they had already been restricted. In view of the given right-censoring, the statistics represent an underestimation of the true number of days.

With respect to the multivariate analysis using logistic regression, we excluded the probably infected athletes (n=56). The model containing five independent variables was significant, χ^2^=27.708, N=981, df=5, p≤0.001, R^2^ (Nagelkerke)=0.048. The results remained robust when the probably infected athletes were included and grouped with first the non-infected and then the infected athletes. Considering demographics and sport categories, male athletes had significantly higher odds of having a SARS-CoV-2 infection than female athletes (OR=1.90; 95% CI 1.30 to 2.76; p<0.001). Furthermore, athletes in team sports were more likely to be infected than individual sports athletes (OR=1.75; 95% CI 1.21 to 2.54; p=0.003), whereas older athletes were less likely to be infected than young athletes (OR=0.97; 95% CI 0.95 to 1.00; p=0.031). Participation in winter sports (OR=1.04; 95% CI 0.69 to 1.58; p=0.849) and training volume (OR=1.03; 95% CI 1.00 to 1.05; p=0.058) did not affect the likelihood of infection.

In March 2021, 68% of the sample indicated a willingness to be vaccinated as soon as they were offered an opportunity (see table 3). There is no significant difference between men and women, χ^2^(1)=0.80, p=0.789, N=1037, Cramer’s V=0.01, and between infected, probably infected and non-infected athletes, χ^2^(2)=4.77, p=0.092, N=1037 Cramer’s V=0.07.

**Table 3:** COVID-19 vaccination willingness of Swiss elite athletes by gender

Willingness to vaccinate	Female athletes		Male athletes		Overall	
	n	%	n	%	n	%
Yes	302	68.2	400	67.3	702	67.7
No	141	31.8	194	32.7	335	32.3
Overall	443	100.0	594	100.0	1037	100.0

The athletes were asked if they are going to get vaccinated against COVID-19 when they are offered vaccination.

## Discussion

The present study investigated SARS-CoV-2 infections in Swiss elite athletes. In the first year of the pandemic, 14.6% of all Swiss elite athletes had been infected with SARS-CoV-2 and 5.4% suspected that they had been infected. Male athletes, young athletes and athletes in team sports had an increased probability of contracting COVID-19. Thus, the overall number of positive tests in the athlete sample within 1 year after the outbreak of the pandemic was considerably higher than in the Swiss population. One possible explanation is that they had more social contacts due to their sporting involvement. The increased probability of contracting COVID-19 in team sports supports this hypothesis. However, it can be assumed that athletes were tested sooner and more often than a person from the general population when experiencing symptoms. In fact, the seroprevalence^5^ for the same period in the Swiss population indicates that the actual spread of the virus was significantly greater than the number of positive tests.^4^

Moreover, a high degree of heterogeneity was found with regards to symptoms severity, the influence on performance and the duration of negative effects.

One year after the outbreak of the pandemic, 68% of the athletes wanted to be vaccinated. This means that the willingness for vaccination was slightly higher compared with the similar age group (25–34 years) of the Swiss population (62%).^9^ At that time, there was no particular pressure yet to be vaccinated in order to participate in competitions. However, it can be assumed that the vaccination rate among athletes was elevated subsequently due to the various awareness campaigns as well as travel and competition regulations that were introduced.

The main limitations of this study are that SARS-CoV-2 infection was self-reported and that there is no information on the type of test (eg, PCR, antigen, serological test) nor the exact timing of the positive test. This also makes it difficult to compare the results with the general population. Furthermore, given the retrospective nature of the study, memory bias could have influenced the reported symptomatology. Future studies should, therefore, use a more detailed survey method to estimate the period prevalence in elite athletes and use a non-athletic control group. Moreover, it would be interesting to examine the cases with long-lasting symptoms more closely, as the impact of long COVID on performance can be lingering. Finally, it remains unclear whether the sample is representative for the population with regard to SARS-CoV-2 infection.
